# Monitoring and Optimization of *Cupuaçu* Seed Fermentation, Drying and Storage Processes

**DOI:** 10.3390/microorganisms8091314

**Published:** 2020-08-28

**Authors:** Joana M. L. Souza, João M. Rocha, Cleísa B. C. Cartaxo, Marcus A. M. Vasconcelos, Virginia S. Álvares, Matheus M. Nascimento, Renata T. B. Yomura, Simara Kaefer

**Affiliations:** 1 EMBRAPA Acre—Empresa Brasileira de Pesquisa Agropecuária, Departamento de Pesquisa e Desenvolvimento, Laboratório de Tecnologia de Alimentos, Acre CEP: 69908-970 Rio Branco, Brazil; joana.leite-souza@embrapa.br (J.M.L.S.); cleisa.cartaxo@embrapa.br (C.B.C.C.); marcus.vasconcelos@embrapa.br (M.A.M.V.); virginia.alvares@embrapa.br (V.S.Á.); renata.beltrao@embrapa.br (R.T.B.Y.); 2 REQUIMTE—Rede de Química e Tecnologia, Laboratório de Química Verde (LAQV), Departamento de Química e Bioquímica, Faculdade de Ciências da Universidade do Porto (FCUP), Rua do Campo Alegre, s/n. P-4169-007 Porto, Portugal; 3 Universidade Federal do Acre, Centro de Ciências Biológicas e da Natureza, Laboratório de Fitotecnia, Br 364, km 04, Bairro Distrito Industrial s/n, CEP: 69915-900 Rio Branco, Brazil; matheusxmattos@gmail.com; 4 Instituto Federal de Educação, Ciência e Tecnologia de Rondônia, Departamento de Pesquisa, Inovação e Pós-graduação, Campus Porto Velho Zona Norte, BR-364, Km 1071, Distrito de Nova Califórnia CEP: 76.848-000 Porto Velho, Brazil; simara-keva@hotmail.com

**Keywords:** autochthonous tropical fruits, cupulate^®^ (product similar to chocolate), *Theobroma grandiflorum* schum, fermentation, drying, storage, project design, microbiology and physiochemical parameters, food heritage, Amazon (Brazil), health promoting, cocoa substitute, cupuaçu pulp and seeds, biotechnology

## Abstract

Cupuaçu [*Theobroma grandiflorum* (Wild ex Spreng.) K. Schum] seeds have been employed for a long time in the Amazon region for food purposes. Similar to cocoa, processed cupuaçu pulp and seeds can be used to produce juices, ice creams, confectionary products and cupulate^®^, which is a similar product to chocolate. However, its market penetration requires the mastery of all processing stages to improve the food quality and safety and to make possible an efficient technology transfer to the local small farmers and communities. Based on the above, the current research work aimed at monitoring and optimizing the consecutive fermentation and drying processes of cupuaçu seeds over 7 days each, as well as storage for 90 days. A greenhouse structure incorporating the fermenter and solar drying terrace was designed to be inexpensive, versatile, easily scalable, and easy to maintain and operate by the local small farmers after a short period of training. This research effort also aimed at giving a vision for the future creation of an integrative and sustainable cupuaçu system covering the economic, social, cultural and environmental vectors. The experimental design comprised 5 batches of 100 kg of seeds each. Several microbiological and physicochemical parameters were performed and correlated with processing variables. Microbiological parameters encompassed viable counts of mesophilic microorganisms, coliforms, yeasts, and molds, whereas physicochemical measures included fermentation and drying temperature, pH, acidity, dry matter, ashes, water activity, color, total proteins, lipids and carbohydrates, and energy. The average seed fermentation temperature varied from ca. 28 to 44 °C, reaching the maximum on day 3 and a final value of ca. 31 °C. Regarding solar drying, the average seed temperatures ranged from ca. 24 °C (at the end) to 39 °C on day 3, and an initial value of ca. 29 °C. The average final seed pH value of drying was 5.34 and was kept during storage. During storage, results demonstrated the existence of significant correlations among several experimental parameters under scrutiny. Finally, bean viable counts obtained during storage unfolded acceptable values of total mesophilic bacteria well below the maximum limit. Viable counts of yeast and molds were generally found between 3 and 4 log(CFU/g_sample_), and total coliforms were also detected, although both were at acceptable levels and well beneath the established maximum limits for food safety.

## 1. Introduction

**Figure 1:**
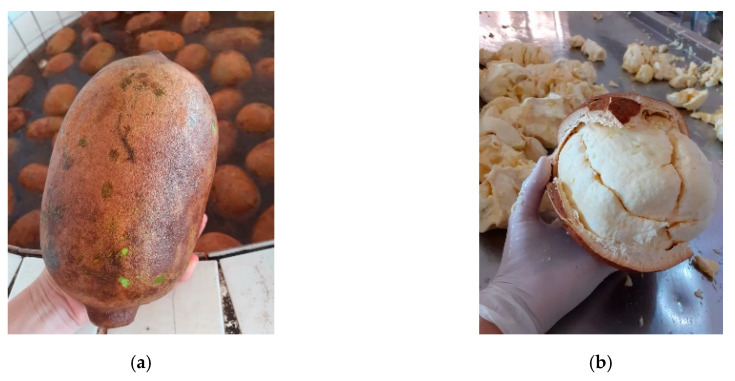
Picture of Cupuaçu (*Theobroma grandiflorum*) (**a**) pods and (**b**) fresh pulp. Courtesy of authors S.K. (Project RECA) and J.M.L.S. (Embrapa), Rio Branco, Acre, Brazil.

Cupuaçu [*Theobroma grandiflorum* (Wild ex Spreng) K. Schum] (reads “cupuassu”) is an Amazonian plant that has shown a significant expansion in the tropical fruit pulp market in Brazil and somehow worldwide (Figure 1).

The largest distribution of the cupuaçu market is located in the Brazilian North region with a presence of 76.7% of the total agricultural establishments that grow this fruit in the country, followed by the northeast region (present in 22.3% of the regional commercial establishments), and only a value as low as 0.8% of cupuaçu farms are located at the Brazilian central west region. Regarding the profile of the producers, they are essentially small farmers, comprising on average as much as 84% of all the cupuaçu establishments visited in 2017. Such figures are important because they set guidelines for the development of environmentally sustainable cupuaçu-based businesses. Among the Brazilian federal states with records of occurrence, Amazonas (34.8%), Pará (27.7%) and Bahia (21.8%) comprise the highest percentages of production in the entire country, representing 84.3% of the national production [1].

The value of cupuaçu production in Brazil in 2017 was R$ 54.8 million (Brazilian Real, BRL) (*ca*. 9.2 M€ in average exchange rate of 2020), of which R$ 41.4 million was produced in the north region and R$ 11.2 million was the estimated production value for the northeast region, leaving only over 2.1 million to be distributed between the Midwest and Southeast regions [1]. The inestimable economic value of this endogenous fruit is based on the industrialization and commercialization of the pulp, which is much appreciated in juices, creams, ice creams, paste sweets, pastry recipes (e.g., cakes and biscuits), among many other forms of consumption and industrial applications [2]. However, due to the actual high demand for tropical fruits and fruit-based products, the agro-food industry has been looking for solutions to the use and valorization of cupuaçu seeds, which are highly rich in fats, proteins, minerals, bioactive compounds, and many other nutrients but which often are discarded and ending up as waste despite these nutritional and health attributes and the high agro-industrial potential. The fruit has high yields of processing, and all parts of the cupuaçu pods can have a useful application. The pulp can be used for the preparation of various food products, the placenta (the seed receptacle) can be used in the preparation of fertilizers, the pod husk/shell can be converted into handmade packaging, whereas the seeds are employed for the production of the aforementioned cupulate^®^, which is a product technologically similar to chocolate [3,4,5].

Previous successful research studies addressing the seed fermentation and drying processes allowed the public research company EMBRAPA—Empresa Brasileira de Pesquisa Agropecuária (Brazilian Agricultural Research Corporation)—to obtain cupuaçu liqueur and cake with characteristics similar to cocoa liqueur and cake, respectively, thus enabling its use in the food industry for the formulation of new products similar to cocoa chocolate and others [6,7,8]. The cupulate^®^ (agglutination of cupuaçu and chocolate) is a good example of a product manufactured from cupuaçu seeds similar to chocolate but with a unique taste, aroma, and texture that is very appreciated in Brazil. Indeed, these autochthonous seeds have high potential to replace/substitute cocoa in food recipes and, consequently, its use is attracting great commercial interest. Nevertheless, its implementation in the market will certainly require more knowledge to better control the processing and achieve high standards of food quality and safety, so that it can be further approved by the standardization bodies and regulatory authorities.

Other research studies on the fermentation and drying processes of cupuaçu seeds for the production of liquor or fat have dedicated their attention to the physicochemical characterization [9,10,11]. However, these do not study the microbiota and related microbiological safety and quality. The identification of the microbiota prevailing in different stages of fermentation and after drying and storage will permit a better control of the processing stages and eventually enable the selection of yeast and bacterial strains of interest to further design microbial starter cultures. These findings will ultimately contribute to a better and constant quality of cupuaçu beans, as well as help understand the whole process and facilitate the technology transfer. Particularly, the identification and quantification of molds (i.e., filamentous fungi) and mycotoxins is of primary importance to prevent cupuaçu seeds and products therefrom against fungal spoilage during their processing (pulping and further fermentation, drying, and storage of cupuaçu seeds) and, concomitantly, to guarantee the desired food safety. It is well known that some opportunistic fungal species produce dangerous mycotoxins to humans and other animals and potentially cause serious illnesses, in addition to the technological effects such as the formation of off-flavors, discoloration, and rotting that results in economic losses. In fact, fermentation has been used ever since in multiple food matrixes (e.g., wine, beer, bread, cheese, sausages, sauerkraut, etc.) to prevent the growth of pathogenic and spoilage microorganisms and to improve other technological aspects such as digestibility, texture, taste, aroma, shelf life, and the production of bioactive compounds. Food fermentation is an effective process toward food quality and safety through the inhibition of deleterious pathogenic and spoilage filamentous fungi and bacteria—e.g., the Enterobacteriaceae, Pseudomonaceae and other Gram-negative bacteria, and the endospore forming Gram-positive bacteria such as *Bacillus* spp. and *Clostridium* spp. Simultaneously, food fermentation enables the growth and exponential multiplication of the competitive and desirable regular non-spore-forming Gram-positive rods and the catalase-negative Gram-positive cocci—for example, lactic acid bacteria (LAB)—such as lactobacilli, pediococci, lactococci, leuconostoc, micrococci, streptococci and group D-enterococci—, and the production by the latter of desirable bioactive, antimicrobial, and antioxidant metabolites, such as small peptides, exopolysaccharides (EPS), organic acids, ethanol, CO_2_, bacteriocins, etc. [12,13,14,15,16,17,18,19,20]. These desirable microorganisms and resulting compounds from their metabolic activity are generally non-toxic and food-grade microorganisms, being classified as Generally Regarded as Safe (GRAS) and Qualified Presumption of Safety (QPS) [15].

The chemical and structural characteristics of cupuaçu are similar to those of cocoa (*Theobroma cacao* L.), in addition to the similarity in the processing stages to obtain the seeds and beans. The process starts when the fruits are opened [21]. It is acknowledged that the control of the natural fermentation process of both cocoa and cupuaçu is complex, and it is indeed at this stage that desirable flavors start to be formed. Even though there are similarities with cocoa, the content and characteristics of cupuaçu seeds are very distinct, both in quantity and quality in sensory perception, acidity, and sugar content [4]. Cupuaçu seed fermentation is not possible without prior pulping. This initial mechanical step can promote significant changes in the endogenous microbiota, which may interfere further with the formation of flavor precursor compounds. The cocoa fermentation process has been studied, and it was demonstrated that a consortium of compatible microorganisms—*viz*. *Saccharomyces cerevisiae* var. *chevalieri*, LAB species (*viz*. *Lactobacillus lactis* and *Lactobacillus plantarum*) and acetic acid bacterial (AAB) species (*viz*. *Acetobacter aceti* and *Gluconobacter oxydans* subsp. *suboxydans*)—acts in synergy during fermentation and play key functions in the processing [21,22,23]. Despite the existence of numerous research studies on cupuaçu seed fermentation, which address the processing per se, the identification of microorganisms presents in the fermented seeds and the effect of seed decortication on the final quality of fermented seeds requires further studies intended at allowing scale-up, evaluating and attaining controlled fermentation methods, increasing cupuaçu bean storage time, and evaluating the capacity of filamentous fungi to produce mycotoxins [8,24,25,26,27,28]. These activities are already foreseen in future projects from the same research group.

Based on the above, the current research work aimed at optimizing and better monitoring and controlling the fermentation, drying, and storage processes of cupuaçu seeds, using low-cost and flexible small-scale processing plants targeted for the local family-based small farmers, having in mind that the household production prevails in the market. These small farmers employ the best agronomical practices and are seen as protective elements of the environment, natural resources, plant genetic heritage, and territorial cohesion. This research effort also aims at valorizing the cupuaçu production and processing within an integrative and broader vision of the economic, social, cultural, and environmental sustainability standpoints. For this reason, theoretical considerations are given in this manuscript to emphasize the importance of those aspects as a whole in future technological transfers. The improvement and control of the processing of cupuaçu made in these research efforts is expected to contribute to diversify the exploitation of autochthonous plants highly appreciated by consumers, who are aware of the importance to strive for natural, nutritious, low-processed and healthy food. This work and an upcoming one are also likely to contribute to the integration of the small farmers into the agro-food chain, namely agriculture production, industrial, and trade chains.

## 2. Materials and Methods

### 2.1. Sampling and Cupuaçu Seed Fermentation and Drying

**Figure 2:**
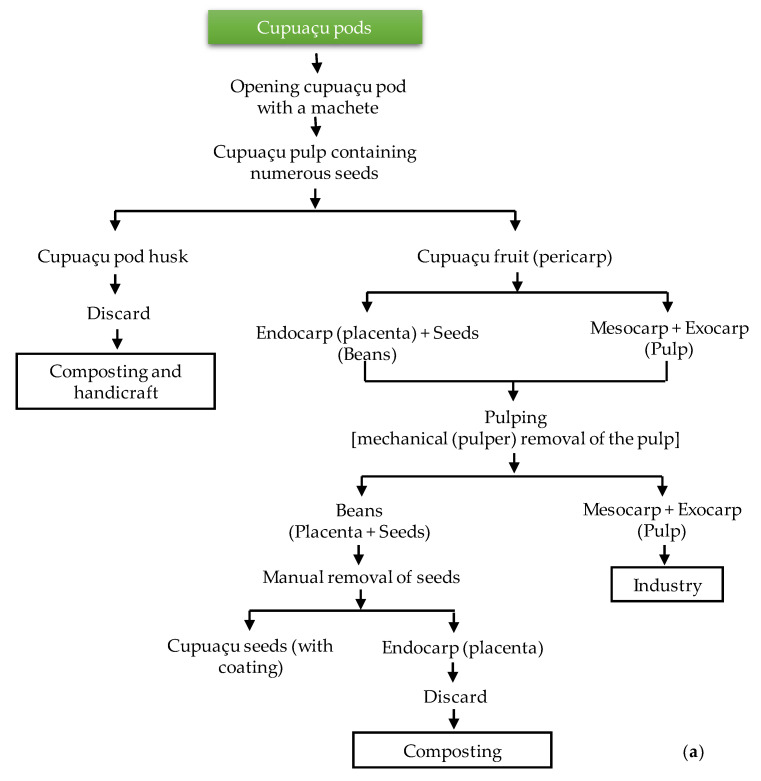

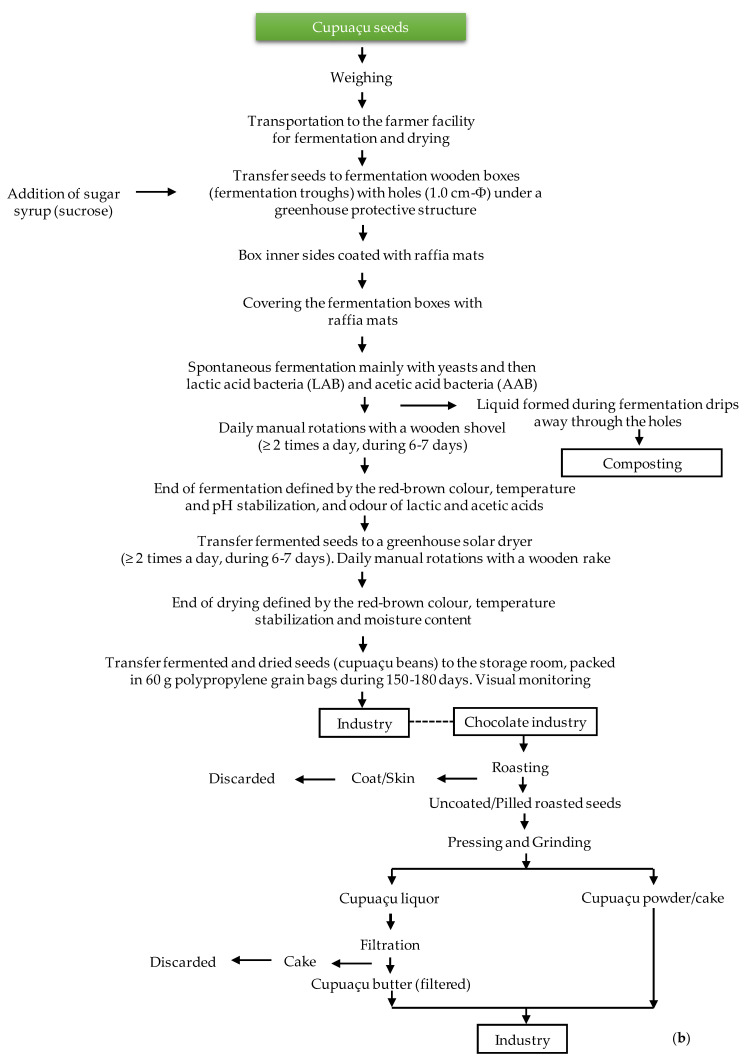
Flowchart for the production of cupuaçu (**a**) seeds and (**b**) fermented and dried seeds with coating (skin).

The flowchart to obtain fermented and dried cupuaçu seeds with coating (skin) is displayed in Figure 2.

Cupuaçu seeds with coating obtained after fruit pulping (mechanical removal of the pulp with a pulper) in the agro-food company of the RECA project (Economic, Intercropped and Dense Reforestation), located at BR-364 road, kilometer (km) 1.071, district of Nova California/Porto Velho, state of Rondônia (RO), Brazil, were transported to Embrapa Acre, in Rio Branco, state of Acre (AC). The RECA Project also comprises an association of small farming families in the state of Rondônia who provided all the cupuaçu samples.

**Figure 3:**
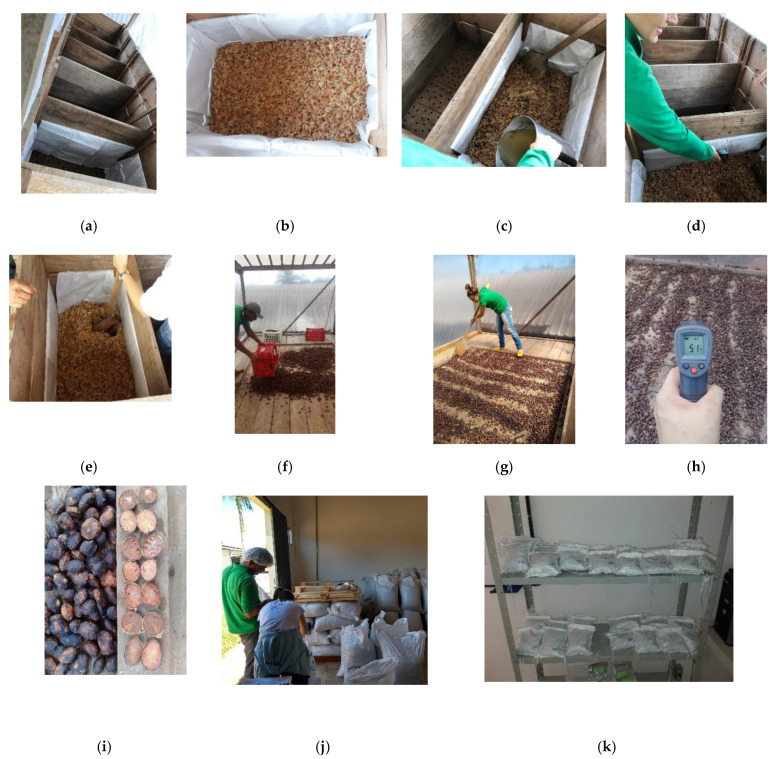
Pictures of Cupuaçu (*Theobroma grandiflorum*) processing: (**a**) Wooden fermenter with 5 compartments (boxes) placed in a greenhouse structure; (**b**) Pulped cupuaçu seeds used for fermentation; (**c**) Adding 30% (*w/w*) aqueous sucrose solution to the seeds in a proportion of 1% (*w/w*); (**d**) Twice daily temperature measurements during the fermentation process; (**e**) Twice daily manual turns of seeds during the fermentation process; (**f**) The same greenhouse structure used to dry the seeds in the solar terrace; (**g**) Twice daily manual turns of beans during the drying process; (**h**) Twice daily temperature measurements during the drying process; (**i**) Cupuaçu beans at the end of the drying process; (**j**) Packing dry beans in 60 kg polypropylene (raffia) grain bags; and (**k**) Dried beans samples packed in 600 g polypropylene (raffia) grain bags for storage studies at the Embrapa facilities. Courtesy of authors J.M.L.S., C.B.C.C., M.A.M.V and S.K. Rio Branco, Acre, Brazil.

Five (*N* = 5) batches (lots) of cupuaçu seeds with coating were submitted independently (and not simultaneously) to the consecutive fermentation and drying processes in the facilities of Acre, in a pilot plant designed and constructed by the research group specifically for these experiments and thought to be flexible and of low cost, aiming at being easily transferred and adapted to the reality of the local small farmers and communities. As a complement to Figure 2, photographs of the cupuaçu processing are depicted in Figure 3. Both the fermenter and solar dryer terrace were part of the same greenhouse structure.

The fermentation was carried out according to the methodology described by Nazaré et al. (1990) [3] in a wooden (free from resins, smells, and off-flavors) fermenter (Figure 3a,b) with 5 compartments (boxes), adding a 30% (*w*/*w*) of aqueous sucrose syrup solution in a ratio of 1% (*w/w*) (i.e., 1 kg of syrup solution per 100 kg of seeds) at the beginning of the fermentation (Figure 3c). The cupuaçu seeds in the compartment were blended twice a day (in the morning and afternoon at the same hour, viz. 10:00 h and 16:00 h, respectively) by manual turns with a wooden shovel (Figure 3e). The fermentation process was undertaken throughout 6–7 days. Temperature measurements were taken twice a day (morning and afternoon) (Figure 3d). After 48 h, seeds were moved daily to the next compartment until the 6th–7th day. The inner sides of the compartments were covered with raffia mats, whereas the bottom had 1-cm ϕ holes to drain liquids (Figure 2 and Figure 3c). The seeds in the fermenter were also covered on the top with the same material.

Following the seed fermentation, a solar drying process was carried out in the same greenhouse structure. The greenhouse solar dryer was a terrace with a wooden floor (Figure 3f). A layer of cupuaçu seeds up to 5–8 cm height was found to be optimal for the drying process. The cupuaçu seeds in the greenhouse terrace were manually revolved with a wooden rake twice a day over 6–7 days (Figure 3g), depending on the progress of the drying process. Similar to fermentation, temperature measurements were also taken twice a day (morning and afternoon) (Figure 3h). The dried cupuaçu beans with coating (skin) (Figure 3i) were packed in 60 kg polypropylene (raffia) grain bags (Figure 3j) and transported to the laboratory, where were homogenized and transferred to a forced air circulation camera (Tecnal TE-394/4, Piracicaba, Brazil) and kept at 50 °C for 24 h or until the moisture content reached up to 8% (*w/w*). Then, the dried beans were packed in 600 g polypropylene (raffia) grain bags (Figure 3k) and kept in a storage room at a temperature of 26 °C and 81% relative humidity (RH) for 150 days. In this work, the storage was studied during the first 90 days. Samplings were performed every 30 days during storage.

Cupuaçu fruit selection was based on visual analysis. Throughout fermentation, drying and storage processing the cupuaçu seeds and beans (i.e., dried seeds) were visually monitored by specialized technicians in terms of evolution of the color, smell, aroma, and detection of possible microbial contaminations. The end of fermentation was defined by the seed color, stability of temperature and pH, and the odor (acetic and lactic acids).

### 2.2. Project Design of the Fermenter, Solar Drier Terrace, and Greenhouse

**Figure 4:**
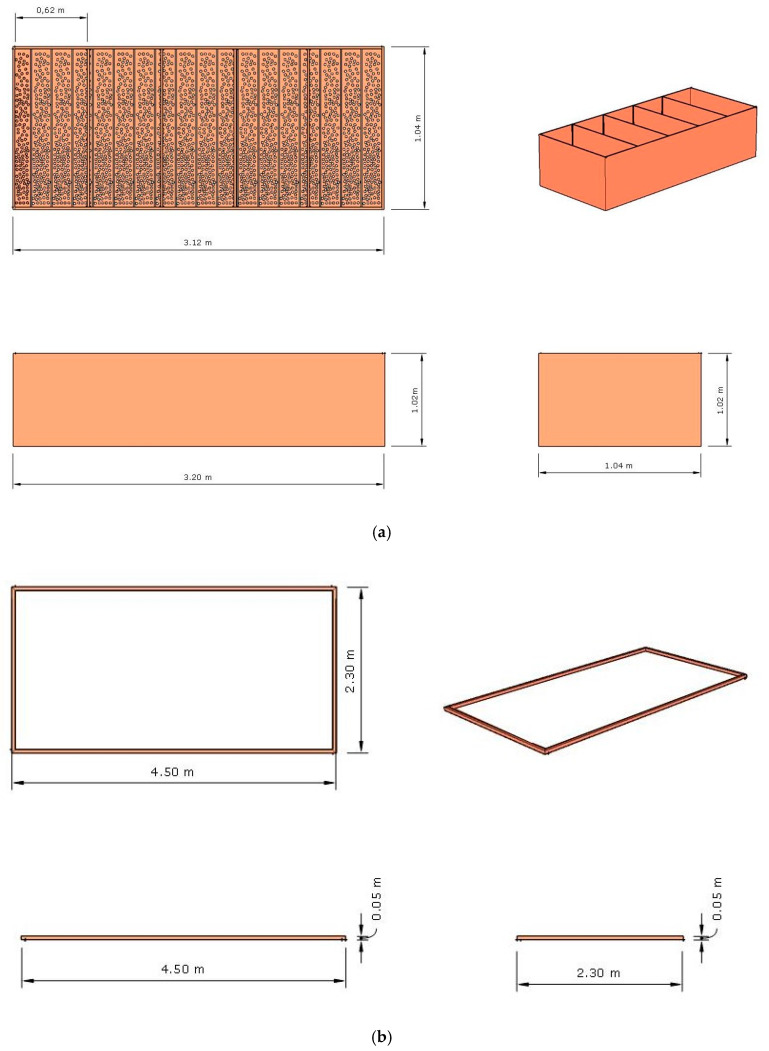

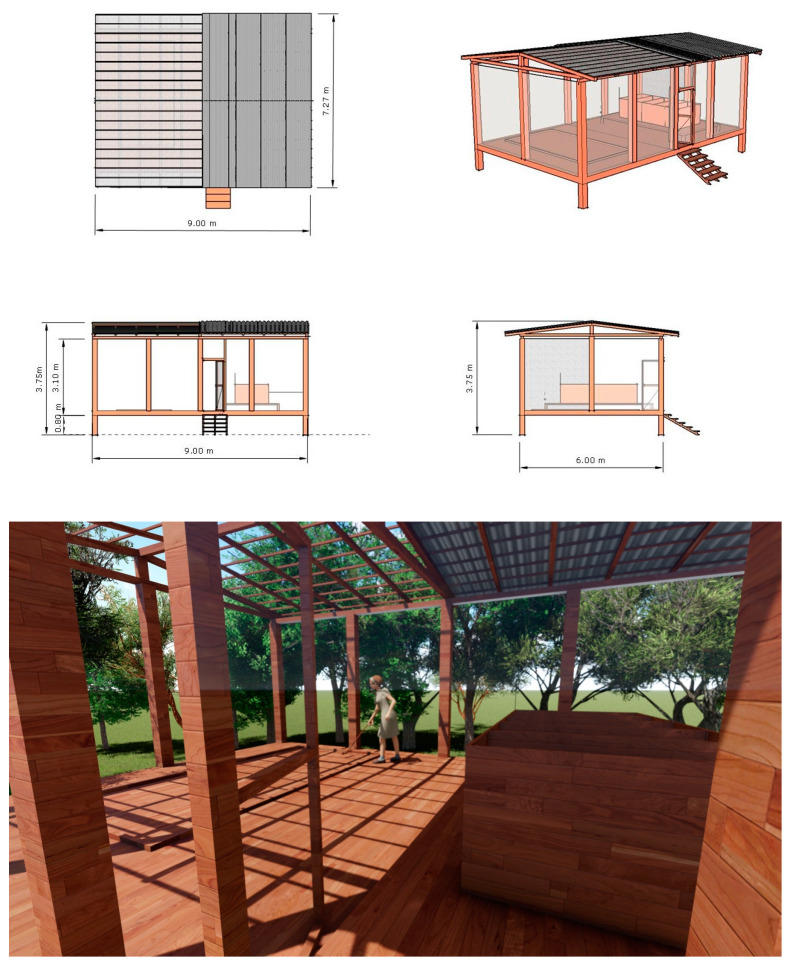

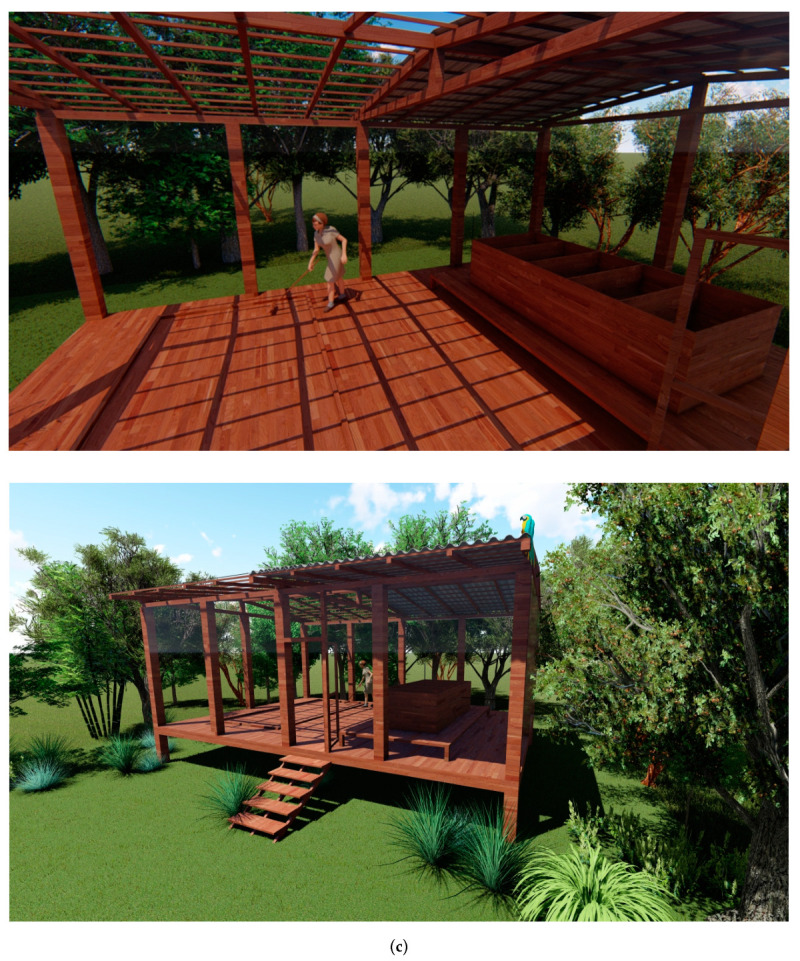
Project design of the (**a**) wood fermenter, (**b**) delimiter/separator of the solar drier terrace made up of four wooden sticks, and (**c**) the suspended greenhouse structure made of wood, side walls of plastic and roof with plastic and cement tiles. The dimensions L × l × h, where “L” is the length, “l” is the width or depth, and “h” is the height were: (**a**) Wood fermenter (3.12 × 1.04 × 1.02) m, each of the five fermenter compartments/boxes (0.62 × 1.04 × 1.02) m, and the bottom of the fermenter with 1-cm ϕ holes to drain liquids; (**b**) Delimiter of the solar drier terrace (2.30 × 4.50) m made up of wooden sticks (l × h) (0.050 × 0.050) m; and (**c**) Greenhouse structure (L × h) (9.0 × 6.0), h and h_ma×_ from the floor to roof of 3.10 and 3.75 mm, respectively, and a greenhouse suspended with four piles of 0.80 m high (from the ground to the floor).

The project design of the pilot fermenter and solar drier terrace placed in a common greenhouse are shown in Figure 4, in complement to Figure 2 and Figure 3. As already mentioned, due to the type of affordable materials chosen and the flexible nature of the design, this pilot greenhouse is easily scaled up and down to better fit with the specific requirements of the local small farmers. The structure is also easy to maintain and operationalize, which is, along with a good knowledge and control of the processing parameters, essential for a successful technological transfer to the local communities.

In this experiment, the fermenter (Figure 4) has five compartments, and the five batches occurred in different time periods. Nevertheless, by increasing the number of compartments of the fermenter and/or creating replicates of the fermenter in series, the throughput can be significantly increased to better fit with specific requirements. Having in mind that the fermentation takes 7 days (day 0 to 7) and the fermenter in this work has five compartments (boxes), in each batch, the seeds to be fermented were kept 2 days (48 h) in the first compartment and then changed to the next one on a daily basis. Hence, this fermenter could receive a new 100 kg batch every two days, and the throughputs would be in this case: 7 days, 100 kg (1 batch); 9 days, 200 kg (2 batches); 11 days, 300 kg (3 batches); 13 days, 400 kg (4 batches); 15 days, 500 kg (5 batches), and so on. Regarding the solar drier terrace (Figure 4), a flexible rectangular separator made up of four wooden sticks [(5 × 5) cm, depth × height] was created to better delimit the required area for drying in a seed layer of up to 5 cm height. This area was enough for independent batch experiments in this research work. Since the total area of the terrace is larger than the delimited area, it can be easily increased (see Figure 3f,g and Figure 4). Conversely, the total drying area can be easily scalable even by enlarging the same greenhouse without the need to build a new greenhouse.

Some important factors to take into account to scale a greenhouse and choose eventually other materials comprise, among others, the desired throughputs, the sun and wind exposition—which influences the fermentation and to a greater extent the drying process, the intensity and average frequency of rain with a direct impact on the maintenance of the structure and in the fermentation and drying processes, the existence of trees and other vegetation with impact on the sun and wind expositions, and the nature and slope of the ground. The greenhouse was made with wood, and a plastic cover was adopted as protection (Figure 3f,g). Accordingly, the greenhouse has a limited robustness, which depends on where is placed as previously referred. Small farmers will be advised to choose alternative inexpensive materials to the malleable plastic to cover the greenhouse whenever possible. The four side walls of the greenhouse are made of plastic.

As can be seen in Figure 4, the pilot greenhouse structure is suspended to prevent atmospheric humidity and rainwater drainage, to facilitate fermenter handling and transfer operations to the drier terrace, as well as to prevent animals from entering. Finally, over the fermenter, the roof of the greenhouse is made of cement tiles to prevent fermentation from direct solar exposition and high-temperature amplitudes, whereas the roof below the solar drier is made of the same material as the side walls, i.e., plastic.

### 2.3. Sampling and Physicochemical Analysis during Storage

At the beginning of the storage (end of drying) and every 30 days, several physicochemical parameters of cupuaçu beans with coating were analyzed. The moisture content was obtained in an oven with forced air circulation (Tecnal TE-394/4, Piracicaba, Brazil) at 105 °C for 8 h and expressed as mass percentage. Ash content was obtained by incineration in a muffle (Quimis Q-318M, São Paulo, Brazil) at 600 °C and expressed as mass percentage [29]. Total proteins were determined by the micro-Kjeldahl using the Association of Official Analytical Chemists (AOAC) standard methods [30], and the measured total nitrogen converted to the amount in mass percentage (%, weight_analyte_ per weigth_sample_) (%, *w/w*) of proteins using the conversion factor of 6.25 (16% N). Total titratable acidity (TTA) determination followed the AOAC standard method [31] and was expressed as a percentage of citric acid.

Several other physicochemical parameters were assessed. The pH was measured with a portable pH meter (Mettler—EasyPlus Titration, Schwerzenbach, Switzerland) [32]. Water activity (a_w_) was attained by direct reading on a portable water activity meter (Aqualab 4TE, Albufeira, Portugal). The surface color of the seeds with coating was determined by the means of a colorimeter (Minolta CR-5, Osaka, Japan). The CIE 1976 L*a*b* color scale was employed to obtain the parameters L* (lightness), a*[chromaticity (red-green)], and b* [chromaticity (yellow-blue)], after calibration of the CIE lab system against a standard white plate. The CIE 1976 L*a*b* color system was created in a way to match the degree of measured color difference with the degree of the perceived color difference [33]. Resorting the measured color values (L*, a*, b*, and L*standard, a*standard, b*standard), ΔE (total color difference) could be calculated using the following expression (1):

(1) $$\Delta E=\;\sqrt{{\left(\Delta L\right)}^{2}+{\left(\Delta a^{*}\right)}^{2}+{\left(\Delta b^{*}\right)}^{2}}$$

where:

ΔL = L*-L*_standard_, [L* varies from white (100) to black (0)] (2)

Δa* = a*-a*_standard_, [a* varies from green (negative values) to red (positive values)] (3)

Δb* = b*-b*_standard_, [b* varies from blue (negative values) to yellow (positive values)] (4)

and where: *L_standard_ = 97.10, a*_standard_ = 0.05 and b*_standard_ = 1.76.

### 2.4. Sampling and Microbiological Analysis during Storage

Regarding the microbiological studies, total viable counts of mesophilic bacteria were carried out according to the official American Public Health Association (APHA) methods [34] by the spread plate inoculation technique in Plate Count Agar (PCA, Merck, Darmstadt, Germany) culture medium at 30 °C during 24–48 h and expressed as logarithm (log) of total colony-forming units (CFU) per gram of sample. Total mold and yeast viable counts were determined using the methodology described by ICMSF (1995) [35], by the spread plate inoculation method in Yeast Extract Dextrose Chloramphenicol Agar (YEDCA, Merck) culture medium at 30 °C during 72–120 h and expressed as logarithm of colony-forming units per gram of sample.

The analyses of thermotolerant coliforms were performed according to the recommendations of the American Public Health Association (APHA) with slight modifications [36]. Samples were weighed, and serial dilutions (10^−1^ to 10^−6^) were aseptically prepared. For the enumeration of thermotolerant coliforms, the most probable number (MPN) technique was used. The presumptive analysis of coliforms was performed in Sodium Lauryl Sulfate broth (LST, Merck) with incubation at 35 °C for 48 h. The enumeration of thermotolerant coliforms was performed in *Escherichia coli* broth (EC, Merck), with incubation at 45.5 °C for 24 h, and the results expressed in NMP.g^−1^.

All chemical, physicochemical, and microbiological experiments carried out right after the drying period (start time at zero days of storage) were made up to 90 days of storage with sampling every 30 days.

### 2.5. Statistical Analysis

The experiments were conducted using a full randomized design, and all analyses were expressed as the average ± standard deviation (Mean ± STDV). Statistical analyses were performed with the software AGROESTAT (Jaboticabal, São Paulo, Brazil) [37].

Firstly, the raw data were transformed to guarantee the conditions of normality and homoscedasticity. The Box–Cox transformation was estimated and applied as proposed by Hawkins and Weisberg (2017) [38]. The estimated transformations for fermentation and drying temperature throughout time, and for physicochemical parameters throughout storage were, respectively:

(5) $$Y=\frac{{\left[0.5\left(X+\sqrt{X^{2}+45^{2}}\right)\right]}^{2.9999}-1}{2.9999}$$

(6) $$Y=\log_{10}\left[0.5\left(X+\sqrt{X^{2}+1.0^{2}}\right)\right]$$

(7) $$Y=\frac{{\left[0.5\left(X+\sqrt{X^{2}+Z^{2}}\right)\right]}^{W}-1}{W}.$$

In Equation (7), the basis *Z* and the exponent and denominator *W* were, respectively: 0.1 and −0.6620 for pH; 2.6 and 0.5421 for TTA; 0.1 and −0.1514 for a_W_; 0.1 and 2.9999 for moisture, ashes, and total lipids; 10.0 and 0.1556 for total fiber; 0.1 and −1.2074 for total carbohydrates; and 334 and 1.8232 for energy. For ΔE and total proteins, the transformation applied was Equation (6).

In the fermentation and drying temperature throughout time after transformation (5 and 6) (see above), the normality of the residuals (P_Shapiro-Wilk_) obtained [39] were, respectively, 0.2422 and 0.1026. The homoscedasticity (P_Levene_) obtained [40] were respectively, 0.8875 and 0.0665. Moreover, in the physicochemical parameters after transformation (5) or (6) (see above), the P_Shapiro-Wilk_ and P_Levene_ values were, respectively: 0.3224 and 0.9370 for pH; 0.3376 and 0.9487 for TTA; 0.7616 and 0.5962 for a_W_; 0.7852 and 0.6039 for ΔE; 0.1425 and 0.2472 for moisture; 0.9432 and 0.24273 for ashes; 0.3120 and 0.5661 for total proteins; 0.4704 and 0.8868 for total lipids; 0.0849 and 0.8555 for total fiber; 0.5364 and 0.8771 for total carbohydrates; and 0.7871 and 0.8654 for energy.

Based on the above information, there was no evidences to reject the normality of the residuals and homoscedasticity at a *p* value of 0.05 (*p* ≤ 0.05) for the transformed values. Therefore, the statistical analyses could proceed, and the principal and interaction effects were determined through the variance analyses. When F-tests were significant, the averages of the factors were compared by the Tukey post-hoc tests, at 5% probability (*p* ≤ 0.05), for multiple comparisons of mean differences. Tukey post-hoc tests were performed with correction to control the Type-I error.

## 3. Results and Discussion

In this section, the results obtained during fermentation, drying, and storage are presented and discussed.

### 3.1. Cupuaçu Seed Fermentation

**Figure 5:**
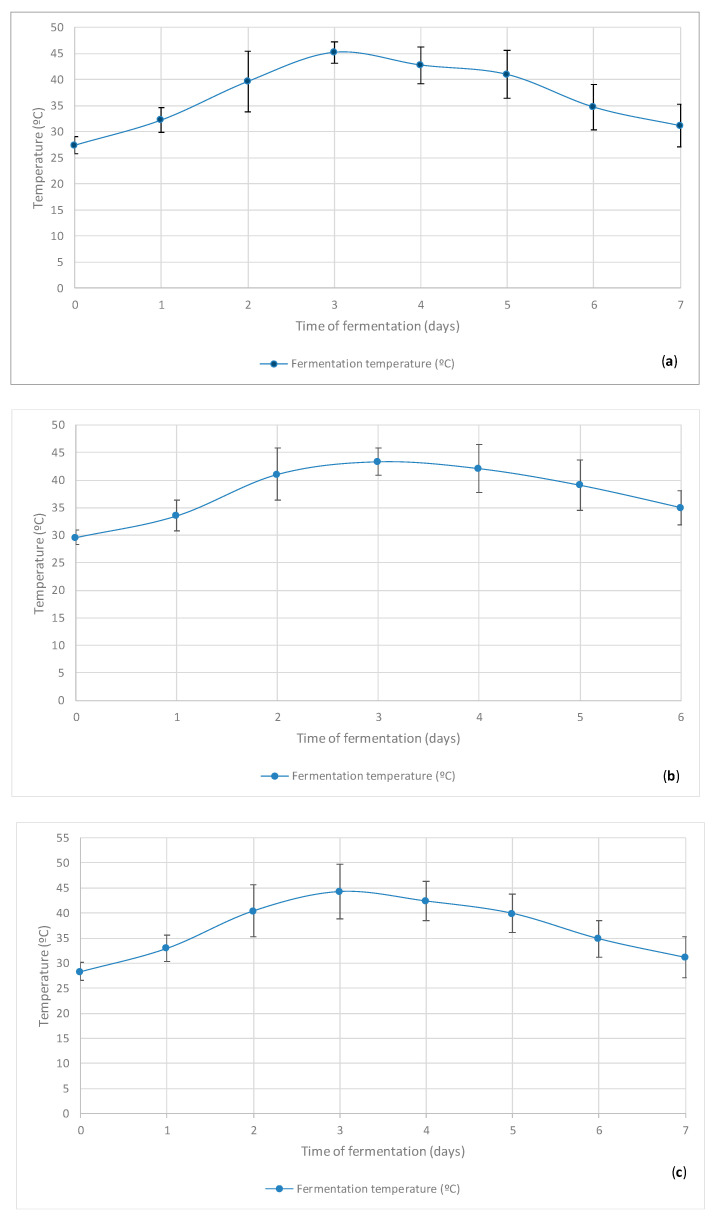
Average values and standard deviations (mean ± STDV) from 3 analytical measures per batches (lot) of 5 lots and 2 sampling periods per each day (morning and afternoon), for the temperature parameters of the seeds throughout 6–7 days of controlled **fermentation of cupuaçu seeds**: (**a**) average in morning; (**b**) average in afternoon; and (**c**) overall average (morning and afternoon). The initial, after fermentation, and after drying weights were, respectively: 100.0, 77.65, and 37.65 kg in lot 1; 100.0, 83.0, and 41.8 kg in lot 2; 100.0, 81.0, and 39.2 kg in lot 3; 100.0, 103.0, and 53.3 in lot 4; and 100.0, 82.0, and 39.5 in lot 5.

The fermentation of cupuaçu seeds and corresponding statistical results are shown below in Figure 5 and Table 1, respectively.

The spontaneous fermentation process is characterized by a rise in the temperature of the cupuaçu seed mass, the conversion of fermentable sugars into ethanol, the conversion of acetic acid from the ethanol, and, in parallel, the production of lactic and citric acids [41]. Monitoring the fermentation (and drying) temperatures in these pilot plants is extremely useful, since it is easily determined and can be used by the local farmers. Simple correlations between temperature and progress of fermentation can also be easily modeled. Besides, resorting to the practical experience of the technicians and local farmers is an efficient way to enable the monitoring and control of the fermentation (and drying and storage) progress. As previously stated, the visual appearance (color and microbial contaminations), smell, aroma, and texture are also important tools to follow up during fermentation.

The fermentation time of cupuaçu seeds was already evaluated by several authors, and it can last from two and a half days (60 h) to 7 days [7,42,43,44]. In the current study, the total fermentation time of the five batches of cupuaçu seeds was evaluated from six to seven days, and the maximum temperatures were generally obtained in day 3, which is in line with the above-mentioned studies. Our results followed similar fermentation time periods as those employed by Vasconcelos (1999) [42], Mattietto (2001) [43], and Cohen and Jackix (2005) [6]. Nonetheless, when evaluating the effectiveness of fermentation processes in pulped and partially pulped (with 7.5% and 15% adhered pulp) cupuaçu seeds in combination with different forms of revolving, Ramos (2020) [45] reported a fermentation time for pulped seeds (a similar condition to the seeds used in our present study) of only 60 h (2.5 half days) against the 6–7 days of our fermentations. Furthermore, when analyzing the formation of volatile compounds during the fermentation of fully pulped cupuaçu seeds, Ramos et al. (2016) [44] found that very short fermentation periods of approximately 60 h were enough to contribute greatly to the development of precursors of desirable flavor traits. Again, these findings are in agreement with the fermentations performed in the current research work.

Throughout the fermentation process, a rise in the average mass temperature was observed, evolving from 28.33 °C on day 0 to a maximum of 44.29 °C on day 3. These results are in agreement with those found by Mattietto (2001) [43], Garcia (2006) [46], and Souza et al. (2016) [8] regarding the required time to reach a maximum temperature during the fermentation of cupuaçu seeds. However, the average maximum temperature observed in this study (44.29 °C) (Figure 5c) was lower than those reported by Mattietto (2001) [43] and Garcia (2006) [46] of 47.0 and 46.7 °C, respectively, but it was higher than that obtained by Souza et al. (2016) [8]. Many environmental conditions are responsible for such a variation—for instance, the ambient temperature, relative humidity, weather conditions, season, use or not of a greenhouse and its materials and designs, raw material, geographic region, number of revolutions, and volume of fermentation seeds, among many other factors. This also means that the optimal fermentation (and drying) conditions are producer-specific, emphasizing the importance to master the variables of the cupuaçu fermentation, drying, and storage processes. Furthermore, Table 1 showed no significant differences in the seed temperatures in day periods of morning, afternoon, and altogether during fermentation. In addition to the greenhouse structure, the use of raffia mats to cover the top and inner sides of the compartments during fermentation is likely to help in maintaining the temperature and favoring a good development of the process.

As explained by Vasconcelos (1999) [42] and Garcia (2006) [46], the increase in seed mass temperature results from the exothermic reactions caused by the growth and multiplication of the spontaneous strictly or facultative aerobic yeast and bacteria microbiota presented therein, namely lactic and acetic acid bacteria among the last microorganisms. This happens due to the aeration promoted by the use of pulped seeds (i.e., seeds separated from the mesocarp), by the daily revolving, by changing from a fermenter compartment to another (which started on the second day of fermentation), and by the liquefaction of pulp residues adhered to the seeds—which flow out through the existing holes at the base of the designed fermenters. There is an expected prevalence of lactic and acetic acid bacteria at the end of the spontaneous fermentation coupled with acid-tolerant yeast strains.

Simultaneously with the fermentation and concomitant temperature rising, there is also the activation and increase of the activity of microbial and seed enzymes, with an emphasis on cellulases, polygalacturonases, invertases, polyphenoloxidases, peroxidases, and lipases, which typically express increased activity up to 72 h of fermentation. The enzymatic activity also plays key roles in the development of precursors for chocolate flavors and aromas [46], and the same is expected to occur with the fermented and dried cupuaçu seeds used in a similar way for the production of cupulate^®^, the “chocolate made of cupuaçu”. It is important to underline that as a result of the rise of temperature during fermentation combined with the formation of organic acids and several other microbial bioactive metabolites, the seed embryo dies, thus losing the ability to germinate [47]. The continuous rise of fermentation temperature (and the resulting physicochemical changes) is also the reason for the loss of microbial and enzymatic activities; thus, it was possible to observe the cascading drop of temperature in the fermenter from the fourth day on, which stabilized on day 7 at 31.18 °C, indicating the end of fermentation.

The spontaneous fermentation process is of the utmost importance toward the reduction of bitterness and astringency of the cupuaçu seeds (for example, by the reduction of solubility of phenolic compounds), the formation of precursors of flavors and aromas (organic and fatty acids, amino acids, alcohols, aldehydes, etc.), as well as the seed preservation during storage and food safety. As already mentioned, monitoring the fermentation and its end moment by farmers is based on the visualization of the development of color, the stabilization of temperature (and sometimes the pH values), and the formation of odors related with the final fermentation stage, such as the lactic and acetic acids.

### 3.2. Cupuaçu Seed Drying

**Figure 6:**
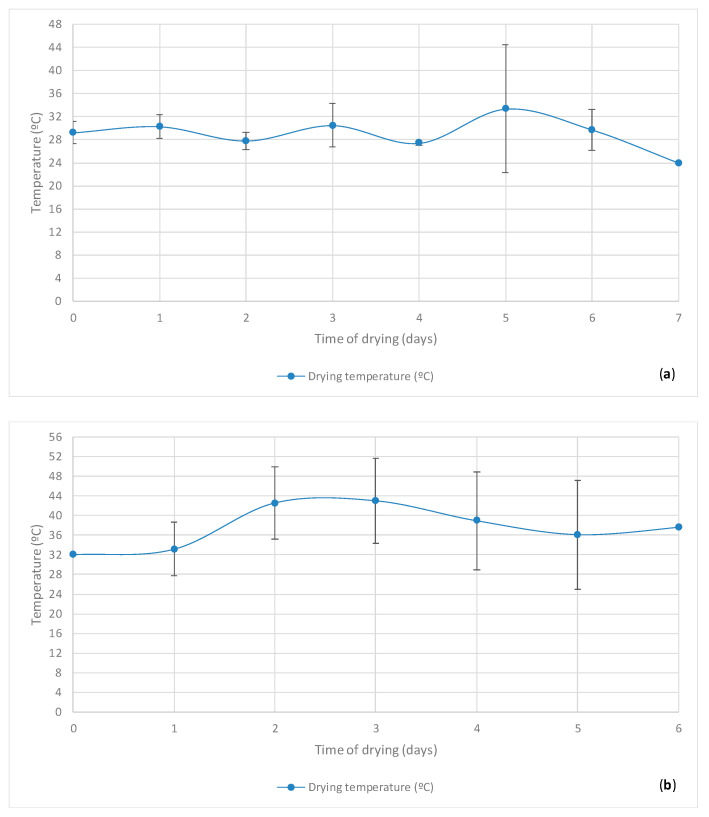

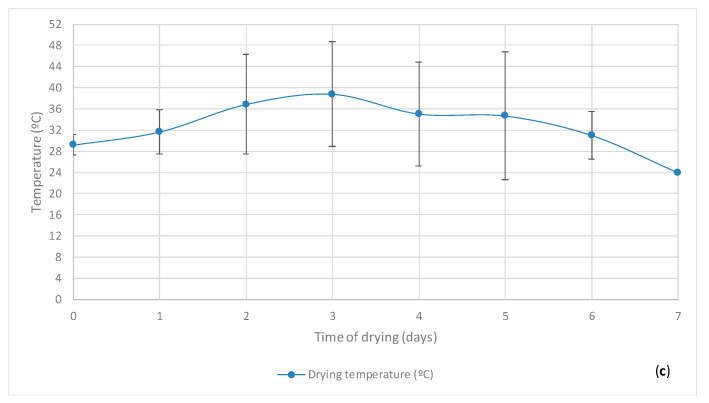
Average values and standard deviations (mean ± STDV) from 3 analytical measures per batches (lot) of 5 lots (batches) and 2 sampling periods per each day (morning and afternoon) for the temperature parameters of the seeds throughout 6–7 days (depending on the evolution of the process) of controlled **drying of cupuaçu seeds**: (**a**) average in morning; (**b**) average in afternoon; and (**c**) overall average (morning and afternoon). The initial, after fermentation, and after drying weights were, respectively: 100.0, 77.65, and 37.65 kg in lot 1; 100.0, 83.0, and 41.8 kg in lot 2; 100.0, 81.0, and 39.2 kg in lot 3; 100.0, 103.0, and 53.3 in lot 4; and 100.0, 82.0, and 39.5 in lot 5.

The drying of cupuaçu seeds and corresponding statistical results are depicted below in Figure 6 and Table 2, respectively.

The drying process was carried out in the same greenhouse covered with plastic to reduce the amplitudes of the tropical temperatures and RHs, among other technical reasons already described in the section Materials and Methods. The average environmental temperature observed during the months of these experiments (February, March, and April 2018) was 25.92 °C, and the average RH was 81% [48,49,50]. The drying process is dependent on natural environmental variables, and its objective is to reduce the seeds moisture to values between 5% and 8% (*w/w*) in a cost-effective manner before packaging and storing, thus preventing mold infestation and allowing the development of chemical reactions that will contribute to the development of desired flavors and aromas. High temperatures are not desirable at the beginning of drying, as the cupuaçu seeds tend to form a drier monolayer on the surface of the skin, limiting the gradual dispersion of moisture into the kernel. On the other hand, milder temperatures can prolong the drying time and favor the appearance of molds in cupuaçu seeds that will promote the appearance of undesirable off-flavors and rotting. Figure 6 and Table 2 show that the average values obtained for the temperature measurements in sampling periods of 2 (48 h), 3 (72 h), 4 (96 h), 5 (120 h), and 6 (144 h) days after the beginning of the drying process presented high amplitudes confirmed by the standard deviations that were not observed in the other time periods.

This geographic region is known for the high temperature and RH amplitudes on the same day. For instance, ambient temperature amplitudes of 23 °C on the same day can be observed. In this context, Table 2 and Figure 6 show detected seed temperatures with an amplitude of 11.52 °C. Therefore, it was inferred that the milder temperatures impaired the necessary drying time, even with the average temperatures in the greenhouse presenting temperature values higher than the environment, which can obviously be attributed to the greenhouse effect. Nevertheless, the experimental data are in agreement with those reported in the work of Banboye et al. (2020) [51], who studied the drying of cocoa in an open space, conventional greenhouse, and modified greenhouse. The drying process and its moisture reduction is fundamental toward the preservation of fermented seeds during storage and to increasing the shelf life and improving the food safety of cupuaçu seeds. The results also demonstrated that the temperatures of the seeds during the day periods of morning and afternoon were significantly different (*p* ≤ 0.05), which is very likely to have a direct impact on the overall drying process. The final pH value of drying (corresponding to the initial pH value of storage) was 5.34.

### 3.3. Physicochemical Analysis during Storage

**Figure 7:**
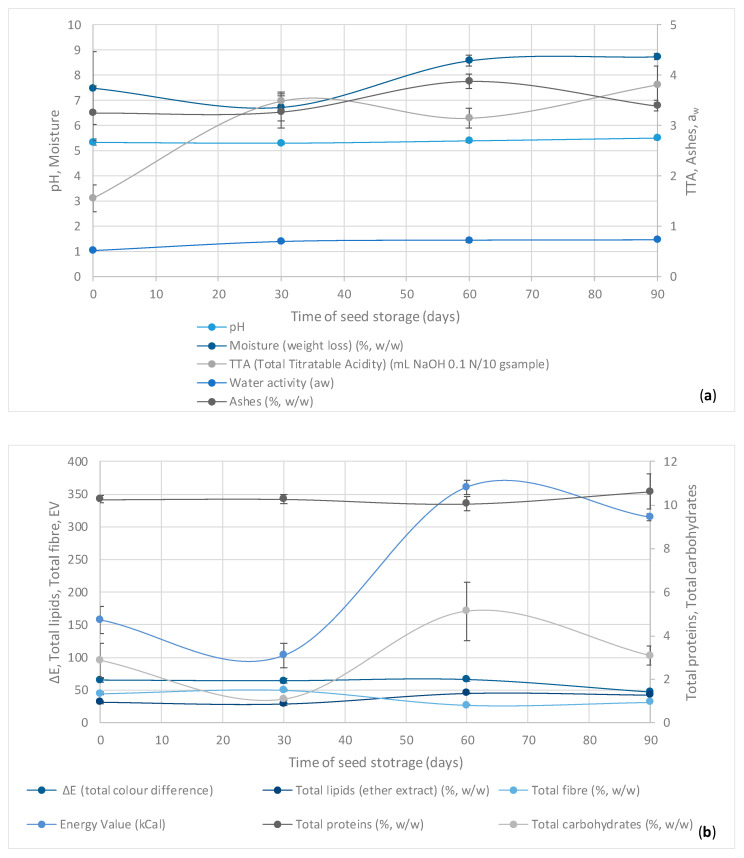
Average values and standard deviations (mean ± STDV) from 3 analytical repetitions for different physicochemical parameters throughout 90 days of **storage of cupuaçu beans**: (**a**) pH, Moisture (weight loss) (%, *w/w*), Total Titratable Acidity (TTA) (mL NaOH 0.1 N/10 g_sample_) and Water activity (a_w_); and (**b**) Total color difference (ΔE), Total lipids (ether extract) (%, *w/w*), Total fiber (%, *w/w*), Total proteins (%, *w/w*), Energy value (%, *w/w*), Total proteins (%, *w/w*), and Total carbohydrates (%, *w/w*).

The physicochemical and corresponding statistical results during storage of cupuaçu beans are given below in Figure 7 and Table 3, respectively.

Oilseed raw materials such as grains, nuts, and almonds have as their major technological barrier the post-harvest processing operations of drying and storage [52]. There is no reliable information on the minimum operational parameters for the drying and storage of fermented cupuaçu seeds and beans, respectively, since the knowledge in this field is still incipient despite its industrial potential and the existing expansion of the culture of cupuaçu plants in several Brazilian states, viz. Amazon and Northeast region, in small- and medium-size agricultural holdings.

In this work, the physicochemical parameters that can be influenced by the storage conditions of fermented and dried cupuaçu beans were evaluated during 90 days with monthly samplings (Figure 7 and Table 3). For the pH value, no statistically significant differences were perceived between sampling over the time. At the beginning of the storage, t_1_ (0 d), the average value was 5.34, and the great average was 5.39. These values show the maintenance of the pH values during storage as a result of the inactivation of the microbiota and enzymes, and they are in agreement with the ones reported by Pereira et al. (2018a) [49] and Santos et al. (2019a, 2019b) [53,54].

Concerning the acidity of fermented and dried cupuaçu seeds during 90 days of storage, there was a clear trend to increase the values from the initial time (t_0_) in relation to the remaining studied times. However, between times t_2_, t_3_, and t_4_, i.e., from 30 days on, no significant differences were observed. The acidity is closely related to the quality and composition of the raw material. Several post-harvest processing operations such as cupuaçu seed fermentation, drying, and/or storage are likely to trigger or inactivate mechanisms of enzymatic hydrolysis and/or oxidation, which almost always changes the concentration of hydrogen ions and the equilibrium balance between proton donators and accepters [55,56]. The behavior of the acidity values over time revealed that the maintenance of lipids present in fermented and dried cupuaçu seeds is closely related to the nature and quality of the raw material, among other factors such as the processing conditions and, most importantly, the conservation/storage conditions. Storage conditions matter because the decomposition of glycerides is accelerated by heating, humidity, and light exposition, while rancidity by hydrolytic, oxidative and microbial mechanisms is almost always accompanied by the formation of volatile free short-chain fatty acids (e.g., butyric acid), aldehydes, and ketones [56]. The use of temperatures throughout artificial seed drying above 80 °C may have severe negative effects on the quality of cupuaçu butter.

The water activity (a_w_) also tended to increase during storage with the mean values at t_1_ (0 d) being the lowest (0.52) and significantly different from the others, which from time t_2_ (30 d) up to t_4_ (90 d) ranged from 0.69 to 0.72, respectively, and did not show any statistically significant difference. This a_w_ profile is paradigmatic in displaying the importance of the drying process to obtain well-developed cupuaçu beans. The a_w_ increase may be the result of the high RH values and the type of packing used. However, considering the stability, products with a_w_ below 0.5 are considered to be very stable.

**Figure 8:**
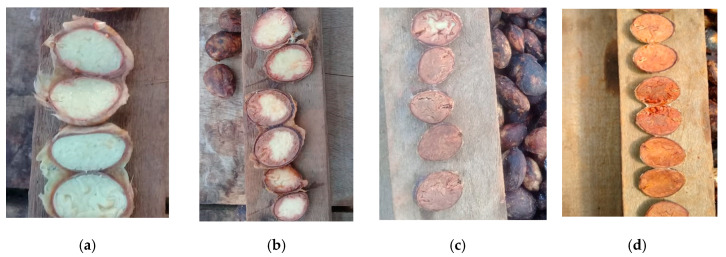
Pictures displaying the color evolution of (**a**) freshly pulped seeds, (**b**) on the third day of fermentation, (**c**) from the third day of fermentation on, and (**d**) fermented and dried beans. Courtesy of the authors J.M.L.S., C.B.C.C., S.K. and M.A.M.V. Rio Branco, Acre, Brazil.

The typical color of cupuaçu seeds before fermentation is cream-yellow (Figure 8a). In the initial stages of fermentation, slight changes were observed on the edge of the seeds (Figure 8b) toward the core. From the third day of fermentation, the reddish-brown color fills the entire space with a matte aspect (Figure 8c). The study of color parameters was carried out in the dried beans (Figure 8d).

Pertaining to the color of the fermented and dried cupuaçu seeds stored for 90 d and packed in raffia bags at an average room temperature of 26 °C and 81% relative humidity [53,54], the luminosity/lightness (L) and the coordinates a* (chromaticity, red-green) and b* (chromaticity, yellow-blue) displayed significant differences only in relation to t_4_ (90 d). The higher L values were found as the storage time increased, thus indicating that the seed coloration was becoming lighter, and it was naturally affected by the type of packaging material used, which allowed the cupuaçu beans to come in contact with ambient air and light. The coordinates a* and b* are correlated with the sample color. The coordinate a*, which indicates a trend toward the darker red color observed in the cupuaçu seeds, disclosed higher values at the beginning of storage, demonstrating that the seed peel or coating is technologically positive by providing an effective protection against oxidation and other mechanisms. In the yellow intensity (b* coordinate), the cupuaçu seeds exhibited the highest values as well at the beginning of storage, where an approximate reddish-brown color indicates a satisfactory fermented cupuaçu seeds. Some factors can affect the color of the seeds, such as the fermentation, drying, and roasting processes [25]. Color is a fundamental attribute for the acceptance of the product by the industry and consumers, providing along with the aroma a pleasant or unpleasant sensation in relation to the food product. The values for L, a*, and b* were close to those reported by Pereira et al. (2018a, 2018b) [49,50] and Souza et al. (2019a, 2019b) [26,27], who were responsible for the initial studies of this research. The values found here were considered acceptable for fermented and dried cupuaçu seeds.

The results of the centesimal composition of fermented and dried cupuaçu beans stored throughout 90 d are shown in Figure 7 and Table 3. Accordingly, statistical significant differences were established between storage times for moisture, (ether extract) lipids, total fibers, carbohydrates, and energy value. Regarding the moisture, it was observed in the early stages of storage (t_0_ and t_1_) average values (*p* ≤ 0.05) below the maximum limit of 8.0% (*w/w*) established by the Concex Resolution n° 160 [57] for cocoa. There is still no specific legislation for fermented and dried cupuaçu seeds. Vasconcelos (1999) [42] mentioned that above 7% (*w*/*w*), cupuaçu beans are vulnerable to mold contamination, with a higher humidity than that found for almonds with film (skin). In agreement with previous data and according to Santos et al. (2019a, 2019b) [53,54], who also carried out some of the initial research activities, the aforementioned recorded environment temperature and RH (26 °C and 81%, respectively) suggest the reason why a moderate increase in the average values of bean moisture were observed in the final time period, viz. t_3_ (60 d) and t_4_ (90 d). These findings were considered close to those reported by Efraim et al. (2010) [58] when studying unroasted nibs from mixtures of cocoa seeds damaged by a disease of cocoa trees caused by the basidiomycete fungus *Moniliophtora perniciosa*, whose moisture ranged between 6.87% and 7.29% (*w*/*w*).

According to the same Figure 7 and Table 3, there were no significant differences in the levels of cupuaçu bean ashes and total proteins during storage. Santos et al. (2019a, 2019b) [53,54] found that the values for ashes and protein content were very similar to the same type of beans produced in the 2018–2019 harvest.

Storage conditions can interfere with the quality of oleaginous raw materials such as fermented cupuaçu seeds and cupuaçu dried beans. Factors such as temperature, relative humidity, storage atmosphere, percentage of broken or malformed seeds, impurities, and the presence of microorganisms, insects, and mites [59] can affect substantially the physicochemical quality such as the total lipid and fiber contents and, consequently, total fiber content and energy value. The storage temperature of cupuaçu beans is one of the most important factors that interferes with the quality, as it can accelerate biochemical and metabolic reactions, by which storage reserves in the supporting tissue are unfolded, transported, and resynthesized in the embryonic axis.

We expected the positive correlations encountered between TTA and ashes as well as the absence of correlation between TTA and pH values, since the latest depends largely on the buffering capacity [17], among other factors. The results also unfolded positive correlations between total lipids, total carbohydrates, and total energy, as well as negative correlations between total lipids and fibers and positive correlations between TTA and total fibers. Unlike what happened during storage, negative correlations between total fibers and carbohydrates might be observed as a result of the microbial, chemical, and enzymatic hydrolyses.

### 3.4. Microbiological Analysis during Storage

The microbiological results of cupuaçu beans during storage are displayed below in Table 4.

The Brazilian National Commission for Food Standards and Standards recommends in the Resolution N° 12/78 a standard maximum of total viable counts on plate agar of 5 × 10^5^ CFU/g_sample_ [or 5.70 log (CFU/g_sample_)] [60]. According to Table 4 (and apart from the presence of countless mesophilic bacteria in the initial and final storage period), one detected an acceptable number of viable counts, since they remained below the established maximum limit. Such an effect results from the physicochemical and microbial changes produced by fermentation and drying processes (as well as during storage), leading to decline of the microbial activity and cell death. It is noteworthy that the aforementioned legislation is not specific for fermented cupuaçu seeds. In this sense, these experimental results are thought to become useful for the standardization bodies to define microbial quality parameters for fermented and dried cupuaçu seeds and further help establish public policies by the policymakers. In fact, such concerted actions are already being taken based on this work.

The presence of yeasts and molds (i.e., non-filamentous and filamentous fungi, respectively) and bacteria in food is natural and may be related to inappropriate handling, contact with equipment, surfaces and utensils not being properly sanitized or, simply, to the contact with the environmental atmosphere, and the same happens with the fermented and dried cupuaçu seeds. The fermentation and drying change the physicochemical and microbiological characteristics of the seeds, preventing microbial contaminations, whereas the non-fermented seeds are more vulnerable to the development of microbial contaminations. However, environmental microbial contaminations may happen at any post-harvest stage. In the case of molds, their deleterious effect is particularly related with the production of mycotoxins during drying and storage stages, which are often harmful for human beings. The number of yeasts and molds depicted in Table 4 unfolded their general presence in values generally between 3 and 4 log (CFU/g_sample_) along the storage period. Although present in relative small and safe viable counts, such results underline the importance to complement these viable counts with the identification of molds and determination of mycotoxins in the future work.

Table 4 also shows the ubiquitous presence of a thermotolerant coliforms group throughout the storage period; however, these are at acceptable levels according to the legislation for the group of foods that encompasses cupuaçu seeds [61], since there is not yet specific legislation for the latter. It was observed that the water activity remained slightly above 0.5 during the entire storage period. The tropical conditions are ideal for the development of unwanted microorganisms, such as the coliforms, which are indicative microorganisms of inadequate hygienic–sanitary conditions that may happen not only during storage but in the entire chain (cultivation, processing, packaging, and transportation of raw materials and products) [62,63]. This group of microorganisms is considered biological hazards, and their occurrence is an indicator of fecal contamination on cupuaçu seeds [64]. Their vanishing during the first days of fermentation is likely due to the abrupt drop of the pH, the formation of organic acids, and other metabolites released by the dominant lactic and acetic acid bacteria and acid-tolerant yeasts, rather than attributable to the storage. In addition to the storage, inadequate hygienic conditions during drying also represent a critical point in the microbial contamination by coliforms. These results during storage emphasize the importance to improve and optimize the processing of cupuaçu seeds and beans to achieve high standards of food safety.

## 4. Conclusions

This research effort aimed at monitoring several microbiological and physicochemical parameters during the fermentation, drying, and storage of cupuaçu seeds as well as the use of flexible and cheap processing plants targeted for the prevalent household production. The experimental results showed an increasing of the fermentation temperatures from 28.33 °C on day 0 to a maximum of 44.29 °C on day 3. This temperature rise is of utmost importance toward the growth of desirable microbiota and activation of endogenous microbial and seed enzymes. Such mechanisms play important roles to develop desirable physicochemical, textural, and sensory traits as well as to achieve the appropriate microbial safety of the seeds and inactivation of seed embryos. The continuous rise of seed temperature results further in the inactivation of such processes. Regarding the drying process, it could be concluded that milder seed temperatures impaired the drying time. Unlike the fermentation, during the drying process, we found significant differences in the seeds between the day periods of morning and afternoon, which unsurprisingly affected the drying process. Furthermore, the monitoring of the physicochemical and microbiological parameters during the 90 d of storage revealed the impacts of the storage process, which was theoretically explained. These results also permitted foreseeing future studies—for example the use of different packaging materials for cupuaçu beans. In order to improve the quality of fermented cupuaçu seeds, it is necessary to carry out further investigations on the microbial and physicochemical dynamics during the fermentation, drying, and storage stages, so as to better control these processes and explore cupuaçu with countless possibilities and potentialities in the agro-food industry. These studies will take place in the scope of complementary research projects in the future.

This work also allowed the design and development of a flexible wood fermenter and solar dryer terrace, which were integrated in the same greenhouse. This system is easily adaptable, scalable, and replicated to the conditions encountered in different local small farms. Thanks to the theoretic and practical/technical experience of the research team from the company Embrapa (Brazilian Agricultural Research Corporation, Brazil), it was possible to gain greater technical and scientific knowledge of the fermentation, drying, and storage processes. As future work, we foresee the determination of other physicochemical, textural, and particularly, microbiological parameters. Another important aspect to be considered in the future is the development of co-cultured microbial starter cultures of yeasts, as well as lactic and acetic acid bacteria (and eventually others, for example propionic bacteria), to better control and standardize the fermentation process, so that high standards of food quality and safety are achieved. Microbial inoculation can also be compared with chemical acidification and spontaneous fermentations. Finally, it will also be essential to develop cost-effectiveness biotechnologies to process and valorize by-products of cupuaçu, which are currently sent for composting. Instead of following composting, these by-products—rich in lignocellulose, polysaccharides and other carbohydrates compounds, among others—may be used to yield high-added value products such as bioethanol, biobutanol, biogas, biopolymers, and biofertilizers, among other possibilities. The microbial processing [anaerobic or solid-state fermentations (STF)] or pyrolysis to release fermentable sugars are promising solutions to further produce single cell proteins (SCP) and oils (SCO) [13,15,20,33,65,66,67,68,69,70,71,72,73,74,75,76]. Cupuaçu endocarp (placenta) could be used to produce vinegar (acetic acid) or as an ingredient in sweets. Such derivate compounds can be re-introduced in almost all type of industrial chains.

Cupuaçu is deemed a natural, healthy, and environmentally friendly tropical fruit with different potential applications in the agro-food industry. The fruit and seeds can be used for juices, ice creams, cupulate^®^ (a “chocolate” made of processed cupuaçu seeds), baking and confectionary, and many other purposes, whereas cupuaçu by-products can be processed and return to new value chains following a regenerative economy approach based on minimum residues generation or zero waste, and 3 or 5 R’s and circular economy policies. As in other food goods, numerous possibilities can be conceived for the best use and valorization of cupuaçu by-products [65,68,74,76,77], which is of primary importance from an economic point of view for the local small farmers, their families, and local communities. Cupuaçu is primarily produced by smallholders who depend considerably on this source of revenue for subsistence. The sustainable production of cupuaçu and the openness to explore their by-products also will help fixating people in such huge rural regions all over the Brazilian cupuaçu producers (e.g., Amazonas, Acre, Pará, Rondônia, Roraima, Amapá, Tocatins, Goiás, Bahia, and other northeast federal regions, etc.)—thus contributing to the economy of the country and preventing the desertification of rural areas.

Cupuaçu and similar autochthonous agro-products are well adapted to the local climate and soil conditions, are typically resilient against climate changes, and have outstanding nutritional and healthy attributes, while promoting a sustainable agriculture and preserving natural resources, plant genetic diversity, and heritage. Indeed, the local small farmers who use the best agronomical practices are seen as fundamental protectors of the environment and biodiversity [13,20,73,78,79], and they are vital to creating discontinuity agricultural stains to prevent forest fires. Such features meet the international and EU policies [80,81,82,83] and particularly, the 2030 United Nations (UN) agenda [84] for taking global actions toward the global sustainability development with the rational use of natural resources.

Enormous challenges are foreseen for the future for improving even more the cost-effectiveness and social and environmental sustainability of cupuaçu production. Exploring plant genetic diversity and breeding lines can improve the productivity and resilience of cupuaçu against the exacerbation of the climate changes and global warming. These measures may help attain sufficient, safe, affordable, nutritious, healthy, and balanced diets—thus responding appropriately to the world population growth—and promote healthy lifestyles. Monitoring cupuaçu production and perhaps implementing precision farming procedures could be considered in the future. The continuous management and technical training of the local farmers and their integration into the agro-food value chain are primordial measures. There are plans to follow the previous and actual research efforts through research projects with national and international intuitions and consortia toward improving the cupuaçu production and processing and transfer the knowledge in loco to the small farmers. Another important challenge is improving and achieving better standard food quality requirements by implementing quality management and food safety systems and product certifications and, simultaneously, being aware of not hampering the expansion of food products manufactured at the farm level to external markets, or even in local markets and food chains. Another important measure is developing new technological cupuaçu-based food products to improve the nutritional quality and the socioeconomic condition of rural population, as well as diversify healthy diets and lifestyles. Furthermore, beyond clean-labeled agro-food commodities, other applications of cupuaçu can be explored, such as natural ingredients (and products therefrom with market high value), cosmetics, and nutraceuticals. However, the economic feasibility still depends on increasing competitiveness through the development of new products and optimizing more efficient processing procedures.

Overall, by providing these measures, it will be possible to attain diversified and healthy diets and lifestyles, territorial cohesion, the socioeconomic development of rural populations, biodiversity and environmental sustainability, and cultural and food heritage preservation. Fresh and low-processed cupuaçu-based foods with implemented quality management and food safety systems meet undoubtedly the requirements of natural, healthy, safety, tasty, sustainable, and authentic food. In addition to the technical support and training qualification of local farmers, the new products and processes are key factors in the direction of integration of local farmers into the whole agro-food supply chain (from production to trading).

Unlike the industrial farming that is turning the forests (including the rainforests) into massive farms and threatening the future of our planet, cupuaçu production is mainly based on small household farming and not on massive monoculture plantations. Cupuaçu local small farmers in Brazil are exemplar concerning their agronomical practices and integration in the surrounding tropical landscapes. Often, they practice tropical intercropping systems, e.g., cacao, coffee, banana, avocado, and Brazil nuts among many other agroforestry examples such as the RECA project that uses integrated agroforestry systems of cupuaçu with peach palm, açaí palm, and others [85]. The use of other plantations is essential to provide the desired light exposition for the proper development of cupuaçu beans. Cupuaçu exploitation should not replicate many of the mistakes seen in the exploitation of cocoa and palm oil, and that is why it is important to look at cupuaçu from a sustainable point of view.

Only through addressing those challenges will it be possible to improve the quality of cupuaçu and similar local products and trigger the integration of local small farmers into agro-food chains from production to the industrial and trade chains. To attain successfully such a goal is fundamental to connecting and facilitating collaboration among all the stakeholders, *viz*. farmers, companies, researchers, governmental and regulatory authorities, policymakers, standardization bodies, consumers, and consumer associations. To integrate the local communities in the whole value chain so as to ensure desirable and fair profits and the required individual livelihoods, industries, trade entities, and consumers must all be called to account. It is fundamental for companies and consumers to practice social and ethical responsibility by fair markets and trade, as well as guarantee direct negotiation and honest payment to the local producers in a win–win philosophy. On this subject, many negative examples can be found all over the world—for example, in the production of palm and coconut oils. A comprehensive traceability of the production and trade of cupuaçu (and any other similar product and system) must be put into practice from “*farm to selling*”. The fair trade of the companies and their social and ethic responsibilities require this traceability and oversight by the policy and regulatory arena, consumers, and general people. Only in this way will it be possible to practice responsible food production consumption, boosting food quality and safety and endorsing an environmental and economic sustainable and resilient domestic-based agriculture with reduced environmental footprints and even with neutral or positive environmental impacts to attain a society with “*zero waste–zero hunger*”.

## Figures and Tables

**Table 1:** Average values and standard deviations (mean ± STDV) from 3 analytical measures per batch (lot) of 5 lots and 2 sampling periods per each day (morning and afternoon) for the temperature parameters of the seeds throughout 6–7 days of controlled **fermentation of cupuaçu seeds**: average in morning; average in afternoon; and overall average (morning and afternoon). The initial, after fermentation, and after drying weights were, respectively: 100.0, 77.65, and 37.65 kg in lot 1; 100.0, 83.0, and 41.8 kg in lot 2; 100.0, 81.0, and 39.2 kg in lot 3; 100.0, 103.0, and 53.3 in lot 4; and 100.0, 82.0, and 39.5 in lot 5. Statistical analysis: F-tests Tukey post-hoc tests at 5% probability (*p* ≤ 0.05) for the multiple comparison of mean differences. Numbers from the same column with the same superscript letter are not significantly different, as obtained by the Tukey’s test (*p* ≤ 0.05). ^NS^ = Not significant; * = Significant difference (*p* ≤ 0.05); ** = Significant difference (*p* ≤ 0.01); CV = Coefficient of variation.

Fermentation Time (days)	Temperature of the Seeds during Fermentation (°C)
Day Period: Morning	
0	27.47 ± 1.62
1	32.28 ± 2.37
2	39.66 ± 5.79
3	45.23 ± 2.02
4	42.79 ± 3.52
5	40.98 ± 4.58
6	34.74 ± 4.32
7	31.18 ± 4.13
Day Period: Afternoon	
0	29.62 ± 1.28
1	33.54 ± 2.81
2	41.06 ± 4.71
3	43.35 ± 2.51
4	42.09 ± 4.34
5	39.09 ± 4.58
6	35.03 ± 3.11
7	---
Day Period: Morning + Afternoon	
0	28.33 ± 1.81
1	32.98 ± 2.65
2	40.44 ± 5.16
3	44.29 ± 5.47
4	42.4 ± 3.94
5	39.93 ± 3.85
6	34.89 ± 3.69
7	31.18 ± 4.13
Statistical Analysis	
Factor	Variance analysis of principal and interaction effects (F test)
Time of fermentation	0.0013 ^NS^
	Mean values ±STDV
Morning	36.79 ± 6.28 ^a^
Afternoon	37.68 ± 5.07 ^a^
Morning + Afternoon	36.80 ± 5.76 ^a^
Overall average	37.9 ± 0.51
CV	27.45

**Table 2:** Average values and standard deviations (mean ± STDV) from 3 analytical measures per batch (lot) of 5 lots and 2 sampling periods per each day (morning and afternoon) for the temperature parameters of the seeds throughout 6–7 days (depending on the evolution of the process) of controlled **drying of cupuaçu seeds**: average in morning; average in afternoon; and overall average (morning and afternoon). The initial, after fermentation, and after drying weights were, respectively: 100.0, 77.65, and 37.65 kg in lot 1; 100.0, 83.0, and 41.8 kg in lot 2; 100.0, 81.0, and 39.2 kg in lot 3; 100.0, 103.0, and 53.3 in lot 4; and 100.0, 82.0, and 39.5 in lot 5. Statistical analysis: F-tests Tukey post-hoc tests at 5% probability (*p* ≤ 0.05) for the multiple comparison of mean differences. Numbers from the same column with the same superscript letter are not significantly different, as obtained by the Tukey’s test (*p* ≤ 0.05). ^NS^ = Not significant; * = Significant difference (*p* ≤ 0.05); ** = Significant difference (*p* ≤ 0.01); CV = Coefficient of variation.

Drying Time(Days)	Temperature of the Seeds during Drying (°C)
Day Period: Morning	
0	29.21 ± 1.92
1	30.26 ± 2.04
2	27.82 ± 1.48
3	30.47 ± 3.72
4	27.4 ± 0.35
5	33.32 ± 11.06
6	29.72 ± 3.51
7	24.0 ± 0.00
Day Period: Afternoon	
0	32.00 ± 0.00
1	33.12 ± 5.45
2	42.54 ± 7.36
3	42.98 ± 8.67
4	38.92 ± 9.99
5	36.08 ± 11.06
6	37.6 ± 0.00
7	---
Day Period: Morning + Afternoon	
0	29.21 ± 1.92
1	31.69 ± 4.16
2	36.88 ± 9.38
3	38.81 ± 10.56
4	35.08 ± 9.78
5	34.70 ± 12.02
6	34.37 ± 11.81
7	24.00 ± 0.00
STATISTICAL Analysis	
Factor	Variance analysis of principal and interaction effects (F test)
Time of drying	6.07 ^NS^
	Mean values ±STDV
Morning	29. 02 ± 2.72 ^b^
Afternoon	40.46 ± 8.67 ^a^
Morning + Afternoon	33.09 ± 4.70 ^a,b^
Overall average	34.19 ± 5.80
CV	4.16

**Table 3:** Average values and standard deviations (mean ± STDV) from 3 analytical repetitions for different physicochemical parameters throughout 90 days of **storage of cupuaçu beans**: pH, Moisture (weight loss) (%, *w/w*), Total Titratable Acidity (TTA) (mL NaOH 0.1 N/10 g_sample_), Water activity (a_w_), the CIE 1976 L*a*b* color scale {L* (lightness), a*[chromaticity (red-green)], and b* [chromaticity (yellow-blue)]}, Total color difference (ΔE), Total lipids (ether extract) (%, *w/w*), Total fiber (%, *w/w*), Total proteins (%, *w/w*), Energy value (%, *w/w*), Total proteins (%, *w/w*), and Total carbohydrates (%, *w/w*). Statistical analysis: F-tests Tukey post-hoc tests at 5% probability (*p* ≤ 0.05) for the multiple comparison of mean differences. Numbers from the same column with the same superscript letter are not significantly different, as obtained by the Tukey’s test (*p* ≤ 0.05). ^NS^ = Not significant; * = Significant difference (*p* ≤ 0.05); ** = Significant difference (*p* ≤ 0.01); CV = Coefficient of variation.

Treatment Time (days) Treatment (t_i_)	pH	TTA (Total Titratable Acidity) (% Citric Acid)	Water Activity (a_w_)	L (Lightness)	a * [Chromaticity (Red-Green)]	b * [Chromaticity (Yellow-Blue)]	ΔE (Total Color Difference)	Moisture (Weight Loss) (%, *w/w*)	Ashes (%, *w/w*)	Total Proteins (%, *w/w*)	Total Lipids (Ether Extract) (%, *w/w*)	Total Fiber (%, *w/w*)	Total Carbohydrates (%, *w/w*)	Energy Value (kCal)
0 (t_1_)	5.34 ± 0.12	1.56 ± 0.27 ^b^	0.53 ± 0.01 ^b^	36.68 ± 4.03 ^b^	15.06 ± 2.17 ^a^	20.72 ± 2.40 ^a^	65.18 ± 2.58 ^a^	7.49 ± 1.45 ^a,b^	3.25 ± 0.55 ^a^	10.26 ± 0.17 ^a^	31.52 ± 2.29 ^c^	44.61 ± 1.96 ^b^	2.87 ± 0.79 ^a^	157.75 ± 21.17 ^c^
30 (t_2_)	5.31 ± 0.04	3.49 ± 0.18 ^a^	0.70 ± 0.02 ^a^	37.42 ± 4.66 ^b^	14.34 ± 1.09 ^a^	20.14 ± 2.29 ^a^	64.15 ± 3.39 ^a^	6.72 ± 0.55 ^b^	3.28 ± 0.32 ^a^	10.27 ± 0.19 ^a^	28.64 ± 1.75 ^c^	50.00 ± 0.90 ^a^	1.09 ± 0.05 ^b^	103.17 ± 18.25 ^d^
60 (t_3_)	5.40 ± 0.07	3.15 ± 0.2 ^a^	0.72 ± 0.03 ^a^	34.63 ± 2.61 ^b^	13.32 ± 0.65 ^a^	18.51 ± 1.26 ^a^	66.05 ± 2.02 ^a^	8.59 ± 0.2 ^a^	3.88 ± 0.14 ^a^	10.05 ± 0.33 ^a^	45.34 ± 0.44 ^a^	27.00 ± 1.06 ^d^	5.13 ± 1.34 ^a^	360.80 ± 11.40 ^a^
90 (t_4_)	5.51 ± 0.06	3.81 ± 0.37 ^a^	0.73 ± 0.01 ^a^	51.11 ± 0.51 ^a^	7.37 ± 0.59 ^b^	4.61 ± 0.15 ^b^	46.65 ± 0.58 ^b^	8.74 ± 0.13 ^a^	3.39 ± 0.11 ^a^	10.62 ± 0.80 ^a^	42.79 ± 0.57 ^b^	31.37 ± 0.43 ^c^	3.09 ± 0.44 ^a^	314.44 ± 5.50 ^b^
Statistical Analysis														
Factor	Variance analysis of the principal and interaction effects (F test)													
Time of storage	5.31 ^NS^	45.25 **	79.40 **	26.74 **	38.86 **	204.98 **	60.53 **	5.48 *	3.26 ^NS^	0.75 ^NS^	177.47 **	248.78 **	94.45 **	262.88 **
Overall average	5.39 ± 0.09	3.00 ± 1.00	0.67 ± 0.09	39.96 ± 7.53	12.52 ± 3.51	15.99 ± 7.65	60.51 ± 9.27	7.88 ±0.95	3.45 ± 0.29	10.30 ± 0.23	37.07 ± 8.23	38.25 ±1 0.84	3.05 ± 1.65	234.04 ± 123.13
CV	0.34	7.28	7.45	20.39	3.70	3.33	0.91	24.15	25,19	1.86	7.86	1.10	9.71	5.45

**Table 4:** Observation values for 3 replicates (R) of the water activity (a_w_), decimal logarithm of total viable mesophilic and yeast and mold counts (colony-forming units) per gram of sample (CFU/g), and most probable number (MPN) of total thermophilic counts per gram (MPN/g) of sample throughout 150 days of **storage of cupuaçu beans** with seed coating.

Treatment (t_i_)– Replicate (R_i_)	Time (Days)	Replicate	Water Activity (a_w_)	Total Mesophilic Viable Counts (log CFU/g)	Total Yeasts and Molds (log CFU/g)	Total Thermophilic Coliforms (MPN/g)
t_1_-R_1_	0	1	0.6437	Countless	3.75	>1100
t_1_-R_2_	0	2	0.6382	Countless	3.80	295.80
t_1_-R_3_	0	3	0.6398	Countless	3.82	357.00
t_2_-R_1_	30	1	0.6745	Countless	3.49	295.80
t_2_-R_2_	30	2	0.6582	Countless	3.73	244.80
t_2_-R_3_	30	3	0.6573	Countless	3.15	153.00
t_3_-R_1_	60	1	0.7207	4.34	5.05	>100
t_3_-R_2_	60	2	0.6435	4.64	4.67	153.00
t_3_-R_3_	60	3	0.6893	4.08	4.28	>1100
t_4_-R_1_	90	1	0.6271	4.66	3.85	>1100
t_4_-R_2_	90	2	0.6297	4.41	3.00	153.00
t_4_-R_3_	90	3	0.6269	4.56	3.90	>1100
t_5_-R_1_	120	1	0.6154	4.04	0.00	>1100
t_5_-R_2_	120	2	0.6241	4.76	3.00	460.00
t_5_-R_3_	120	3	0.6351	4.64	3.90	>1100
t_6_-R_1_	150	1	0.6386	Countless	3.70	120.00
t_6_-R_2_	150	2	0.7029	Countless	3.70	112.20
t_6_-R_3_	150	3	0.6896	Countless	4.58	112.20
